# The complete chloroplast genome of *Prunus mira* koehne (Prunoideae, Rosaceae), a wild and indigenous peach on Tibet, China

**DOI:** 10.1080/23802359.2019.1679048

**Published:** 2019-10-24

**Authors:** Wenquan Bao, Dun Ao, Tana Wuyun, Tiezhu Li, Lin Wang, Huimin Liu

**Affiliations:** a College of Forestry, Inner Mongolia Agricultural University, Hohhot, Inner Mongolia, China;; b College of Grassland, Resources and Environment, Inner Mongolia Agricultural University, Hohhot, Inner Mongolia, China;; c Non-timber Research and Development Center of Chinese Academy of Forestry, Zhengzhou, Henan, China;; d Non-Timber Forest Research and Development Center, Chinese Academy of Forestry, Zhengzhou, Henan, China

**Keywords:** Chloroplast, phylogenetic analysis, *Prunus mira* Koehne, whole genome sequence

## Abstract

*Prunus mira Koehne* belonging to family Rosaceae, is an indigenous species distributed in Tibet, China. *De novo* assembly with low coverage whole genome sequencing data facilitated to generate the complete chloroplast (cp) genome of *P. mira* in this study. The genome was a circular DNA molecule with 158,153 bp in length. It exhibited a typical quadripartite structure comprising a large single-copy region (LSC, 86,319 bp), a small single-copy region (SSC, 19,022 bp) and a pair of inverted repeat regions (IRs, 26,406 bp each). A total of 112 genes were predicted, which included 78 protein-coding genes, 30 tRNA genes, and 4 rRNA genes. Phylogenetic analysis indicated that *P. mira* was the most ancestral and basal lineage within the subgenus *Amygdalus* (Prunoideae subfamily), which is conform to the traditional classification.

*Prunus mira Koehne* (2n = 2x = 16), belonging to Prunoideae, Rosaceae family, is perennial deciduous trees native in China. It is widely distributed at altitudes from 2500 to 3500 m in the Tibetan plateau (Li et al. 2014). This species was recognized as an important genes pool for the germplasm improvement of cultivated peach, and also be utilized for soil erosion control, vegetation restoration and rootstocks for its high tolerance to drought, cold and barren soil (Fang et al. 2008; Hao et al. 2009). To date, considerable efforts have focussed on its ecological (Fang et al. 2008) and genetic diversity analysis (Bortiri et al. 2001). The phylogenetic relationship of *P. mira* and the taxonomical position were always controverted (Mowrey and Werner 1990). In this study, we generated the complete chloroplast genome sequence of *P. mira*, which could provide basic genetic resource and to help us verify the phylogenetic relationship between *P. mira* and its relative species.

The plant material of *P. mira* was obtained from Linzhi, Tibet, China (29°52.4556′ N, 93°58.5343′ E, Altitude 3358 m). The total DNA was extracted from fresh leaves with a modified CTAB protocol. The voucher specimen (BG3 170011) was deposited in the Inner Mongolia agricultural university Herbarium. An Illumina paired-end (PE) library with 500-bp insert size was constructed and sequenced using an Illumina HiSeq 2500 platform (Illumina, San Diego, CA, USA) by Beijing Genomics Institute (BGI-Shenzhen). After quality trimming, a total of 1.16 Gb clean PE reads (Phred scores >20) were assembled into the contigs using SOAP denovo software (Li et al. 2009). Three typical chloroplast contigs were ordered and merged into a single draft sequence compared with the chloroplast sequence of *P. persica* (NC04697) as a reference. Further validation was also performed using manual correction by PE reads mapping. Genome Annotation was performed with Dual Organellar Geno Me Annotator (DOGMA) (Wyman et al. 2004) (http://dogma.ccbb.utexas.edu/) and the annotation result was manually validated by BLAST searches. The similarity of complete chloroplast genome sequence of other 17 Rosales species (*Morus indica* NC008359 as outgroup) was aligned using MAFFT version 5 (Katoh et al. 2005). Phylogenetic tree was generated by maximum likelihood (ML), maximum parsimony (MP), and neighbor-joining (NJ) analysis using MEGA 6.0 (Tamura et al. 2013) (http://www.megasoftware.net/) with 1000 bootstrap replicates.

The complete chloroplast genome of *P. mira* exhibited a circular DNA molecule of 158,153 bp in length, with overall GC content 36.74%. It was separated into a large single copy (LSC) region of 86,319 bp and a small single copy (SSC) region of 19,022 bp by a pair of inverted repeats (IRa and IRb, 26,406 bp). The GC content in IRs regions (42.56%) was higher than LSC (34.60%) and SSC (30.33%). In genome, a total 112 unique coding regions were predicted, comprising 78 protein-coding genes, 30 tRNA genes, and four rRNA genes. In the IRs regions, 19 coding regions were duplicated, giving a total of 131 genes in the whole genome. Among all unique genes, 16 genes contain one intron and two genes (*ycf3* and *clpP*) with two introns. All the coding regions accounted for 57.82% of the whole genome. The genome sequence with complete annotation information was deposited at GenBank database under the accession number KX889393.

Phylogenetic analysis revealed three major groups, representing Prunoideae, Maloideae and Rosoideae subfamily. The monophyly of the genus *Prunus* was well-supported with high bootstrap value (Figure 1). Three subgroups were also detected in genus *Prunus*, displaying the congruent phylogenetic relationship among subgenera (Lee and Wen 2001; Cho et al. 2016). *P. mira* was the most ancestral and basal lineage within the subgenus *Amygdalus*, which was, in turn, a sister to *P. kansuensis* and *P. persica*. This result was congruent with previous studies by isozyme (Mowrey and Werner 1990) and other molecular markers (Wen et al. 2008; Delplancke et al. 2016).

**Figure 1.**
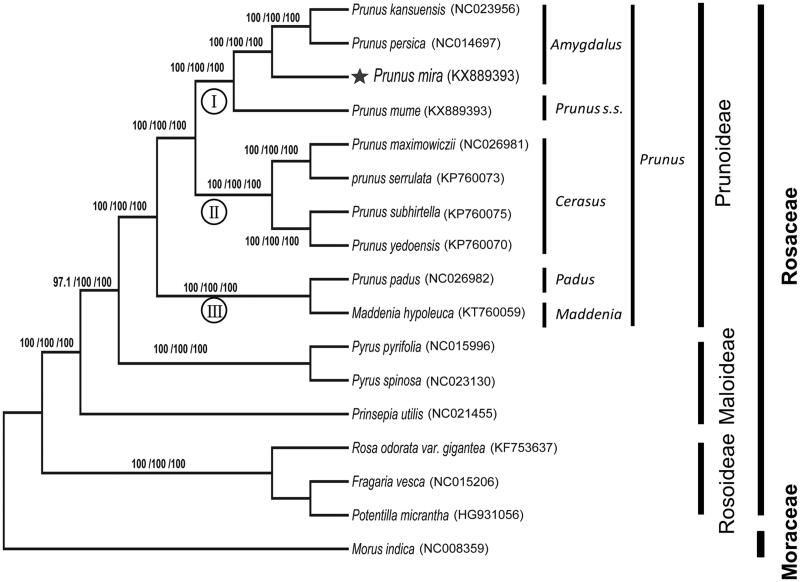
Phylogenetic tree of *Prunus mira* with other 16 species belonging to the Rosales. Tree was inferred from the complete chloroplast genome sequences using the ML method with a GTR model, MP method, and NJ method with a K-2P model. Only the framework of the ML tree was presented. Numbers in the nodes were the bootstrap values from 1000 replicates with an arrangement of ML/MP/NJ methods. Symbol (I,II,III) in the nodes represent three subgroups in genus *Prunus*.
